# Coarse-graining and hybrid methods for efficient simulation of stochastic multi-scale models of tumour growth

**DOI:** 10.1016/j.jcp.2017.09.019

**Published:** 2017-12-01

**Authors:** Roberto de la Cruz, Pilar Guerrero, Juan Calvo, Tomás Alarcón

**Affiliations:** a Centre de Recerca Matemàtica, Edifici C, Campus de Bellaterra, 08193 Bellaterra (Barcelona), Spain; b Departament de Matemàtiques, Universitat Autònoma de Barcelona, 08193 Bellaterra (Barcelona), Spain; c Department of Mathematics, University College London, Gower Street, London WC1E 6BT, UK; d Departmento de Matemática Aplicada, Universidad de Granada, Avda. Fuentenueva s/n, 18071 Granada, Spain; e ICREA, Pg. Lluís Companys 23, 08010 Barcelona, Spain; f Barcelona Graduate School of Mathematics (BGSMath), Barcelona, Spain

**Keywords:** Multi-scale modelling, Hybrid methods, Age-structured model, Reaction–diffusion systems, Tumour growth

## Abstract

The development of hybrid methodologies is of current interest in both multi-scale modelling and stochastic reaction–diffusion systems regarding their applications to biology. We formulate a hybrid method for stochastic multi-scale models of cells populations that extends the remit of existing hybrid methods for reaction–diffusion systems. Such method is developed for a stochastic multi-scale model of tumour growth, i.e. population-dynamical models which account for the effects of intrinsic noise affecting both the number of cells and the intracellular dynamics. In order to formulate this method, we develop a coarse-grained approximation for both the full stochastic model and its mean-field limit. Such approximation involves averaging out the age-structure (which accounts for the multi-scale nature of the model) by assuming that the age distribution of the population settles onto equilibrium very fast. We then couple the coarse-grained mean-field model to the full stochastic multi-scale model. By doing so, within the mean-field region, we are neglecting noise in both cell numbers (population) and their birth rates (structure). This implies that, in addition to the issues that arise in stochastic-reaction diffusion systems, we need to account for the age-structure of the population when attempting to couple both descriptions. We exploit our coarse-graining model so that, within the mean-field region, the age-distribution is in equilibrium and we know its explicit form. This allows us to couple both domains consistently, as upon transference of cells from the mean-field to the stochastic region, we sample the equilibrium age distribution. Furthermore, our method allows us to investigate the effects of intracellular noise, i.e. fluctuations of the birth rate, on collective properties such as travelling wave velocity. We show that the combination of population and birth-rate noise gives rise to large fluctuations of the birth rate in the region at the leading edge of front, which cannot be accounted for by the coarse-grained model. Such fluctuations have non-trivial effects on the wave velocity. Beyond the development of a new hybrid method, we thus conclude that birth-rate fluctuations are central to a quantitatively accurate description of invasive phenomena such as tumour growth.

## Highlights

• A hybrid method for stochastic multi-scale models of cells populations that extends existing hybrid methods for reaction–diffusion system.
• Our analysis unveils non-trivial macroscopic effects triggered by noise at the level of structuring variables.
• Our hybrid method hugely speeds up age-structured SSA simulations while preserving stochastic effects.

## 1 Introduction

Cells behaviour within tissues respond to a number of stimuli. Their behaviour result from a complex network of interactions between genes and gene products which ultimately regulates gene expression. Such systems of gene regulation are often modelled as non-linear, high-dimensional dynamical systems whose structure has been moulded in the course of biological evolution. In addition to such intracellular complex dynamics, cells are also influenced by intricate interactions between different components of the biological systems at all levels, from complex signalling pathways and gene regulatory networks to complex non-local effects where perturbations occur at whole-tissue level [3], [61], [47], [51], [50], [19], [58], [39]. These and other factors contribute towards a highly complex dynamics in biological tissues which is an emergent property of all the layers involved. To tackle such complexity, a number of multi-scale models of biological systems, particularly in the context of tumour growth, have been developed [3], [40], [61], [47], [51], [59], [72], [9], [46], [50], [60], [19], [55], [73], [20], [58], [70], [13], [15], [64], [39], [74].

Many current multi-scale models of tumour growth formulated so far are individual-based, i.e. cells are individually resolved and their response to different types of cues (chemical, mechanical, etc.) is explicitly described by models of cell behaviour of varying levels of complexity [3], [40], [61], [50], [19], [55], [58], [70], [15], [39]. Further to the individual-based approach to multi-scale modelling of biological cell populations, we have recently introduced new stochastic models that allow to analyse the effects of fluctuations, both at the intracellular level (intrinsic noise in signalling pathways and gene regulatory networks) and at the level of the birth-and-death dynamics of cells [32], [17].

Multi-scale approaches have been shown to have both strengths and limitations. Among the latter, it prominently features the computational intensity of these models. The level of detail they involve implies that large scale simulations are computationally costly, which limits the scope of such models. In order to simulate growth in a wider range of conditions, along with model development, algorithms and analytic methods must be developed that enable us for more efficient analysis and simulation of such models. The formulation of hybrid methods for multi-scale models of tumour growth [44], [42], [43] is one such development. The basis of hybrid methodologies is to use models at different resolutions in different regions of the simulation domain, whereby cells (or other structures such as vessels in models of angiogenesis) are individually resolved in some region of interest. Away from such region, the system is described by a lower-resolution, coarse-grained model, obtained for example by means of homogenisation methods [12], [65], [54], [52], [53], [48]. Such homogenised model describes the system at a reduced level of detail but with the benefit of a much smaller computational cost. The challenges involved in these hybrid methodologies include defining criteria to identify the different domains, derive coarse-grained models consistent with their individual-based counterparts, and formulate the appropriate boundary conditions between the individual-based and coarse-grained regions.

A similar situation arises in a different area in which fair progress has been made: stochastic reaction–diffusion systems. Such systems are also costly to simulate using standard methods (i.e. variations of the Gillespie method [68], [6], [21]), so it is often necessary to resort to hybrid methods [49]. The rationale for a hybrid method is that noise levels, roughly associated with the local population or number of particles, is not uniform over the whole system, resulting in regions where fluctuations have more severe effects than in others. An archetypic example of this situation is the propagation of fronts such as travelling waves [7], [8], [49], [14]. In such systems, the population behind the propagating front approaches the carrying capacity of the system. If the carrying capacity is large enough, fluctuations in the region behind the front will be relatively small, so that the system may be described by the mean-field limit of the system. By contrast, at the front and ahead of it fluctuations dominate system behaviour and therefore the full stochastic description is needed. Such inhomogeneities in the noise level have been exploited to formulate hybrid simulation methods. According to this methodology, the mean-field limit of the system is used in low-noise regions which are then coupled to the full stochastic dynamics describing the high-noise regions. The coupling between both descriptions is achieved by means of appropriately defined boundary conditions at the interface(s) between mean-field and stochastic regions [49], [22], [34], [23], [62], [67], [75], [71].

In this paper, we extend and further develop the hybrid method formulated by Spill et al. [67] for stochastic reaction–diffusion systems to stochastic multi-scale models of tumour growth. Such models [32], [17] consider fluctuations regarding both number of cells (*population* noise) and the intracellular (cell-cycle) dynamics (*structure* noise), and consequently any attempt to formulate a hybrid method for such systems must find a way to accommodate both types of noise. Structure noise is associated with noise at the intracellular level and it manifests itself in fluctuations of the birth rate. We show in our analysis that this source of noise is at least as important as the population noise on the behaviour of the system. In particular, we show that the speed of propagation of travelling wave solutions is heavily affected by birth rate fluctuations at the leading edge of the front. More specifically, when a model in which the intracellular dynamics is coarse-grained (i.e. fluctuations of the birth rate are averaged out) is considered, the speed of the travelling wave front is over-estimated by a rather significant percentage. However, when the coarse-grained mean-field model is coupled to the full stochastic multi-scale population-dynamical model, the deviation travelling wave speed is very much rectified and a much more accurate result is obtained. This result demonstrates the usefulness of such hybrid approaches: they can recover accurately the behaviour predicted by the more detailed models whilst, by averaging out some of those details in regions where they are not necessary, their computational performance is much improved.

The paper is organised as follows. In Section 2, we present a summary of the stochastic multi-scale model. For an in-depth presentation, the reader is referred to de la Cruz et al. [17]. Section 3 contains a multiple scale asymptotic analysis which concludes with the derivation of versions of both the full stochastic model and its mean-field limit where the intracellular dynamics (i.e. age-structure) has been coarse-grained. The resulting models are described by the growth rate corresponding to the intracellular state distribution (age-distribution) of the population being at equilibrium (in a sense to be precisely defined in Section 3.2.4). In Section 4, we introduce the hybrid method for the stochastic multi-scale model, which is an extension of that developed by Spill et al. [67] to accommodate the intracellular dynamics (i.e. age-structure) associated with the multi-scale model. In Section 5, we proceed with an assessment of the accuracy of the coarse-grained and hybrid models for travelling wave solutions. We take as a benchmark the full stochastic multi-scale model solved by means of the age-structured Gillespie algorithm [17]. During this analysis we conclude that failure of the coarse-grained growth rate to describe the population dynamics at the leading edge of the front is responsible for the discrepancies between the travelling wave speeds. The hybrid method does away with such discrepancies by providing a more accurate description of the population dynamics within that region. Finally, in Section 6 we discuss our results and present our conclusions.

## 2 Summary of the stochastic multi-scale model

**Fig. 1:**
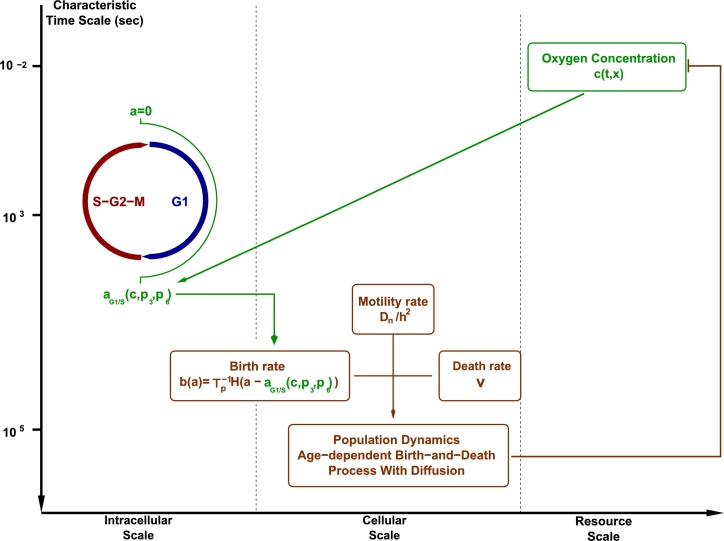
Schematic representation of the different elements that compose our multi-scale model. We show the different levels of biological organisation as well as associated characteristic time scales [32], [17] associated to each of these layers: resource scale, i.e. oxygen which is supplied at a constant rate and consumed by the cell population, cellular scale, i.e. oxygen-regulated cell cycle progression which determines the age-dependent birth rate into the cellular layer, and, finally, the cellular scale, which is associated to the stochastic population dynamics.

Before proceeding further, we present a general overview of the stochastic multi-scale model of tumour growth as well as a summarised discussion of the different elements involved in the formulation of the stochastic multi-scale model. The model presented here is closely related to that presented in [17], the main difference being the introduction of spatial heterogeneities, which were neglected in our previous work. The model we present in this paper accounts for processes with widely different characteristic time scales, as depicted in the scheme shown in Fig. 1. This model intends to describe the growth of cellular populations in a spatially heterogeneous environment under the restriction of finite amount of available resources (in this case, oxygen c(t,x)) supplied at a finite rate, S(t,x).

The general approach to the stochastic population dynamics used here is a natural generalisation of the standard continuous-time birth-and-death Markov process and its description via a Master Equation [24]. de la Cruz et al. [17] showed that the consideration of the multi-scale structure of the system, i.e. the inclusion of the physiological structure associated with the cell-cycle variables, can be accounted by an age-structure within the population: the birth rate depends on the age of cell (i.e. time elapsed since last division) which determines, through the corresponding cell-cycle model, the cell-cycle status of the corresponding cells.

We summarise the different sub-models involved in the formulation or the stochastic multi-scale model schematically represented in Fig. 1. For a detailed discussion of a non-spatial version of the model we refer the reader to de la Cruz et al. [17]. Here, we emphasise the new elements introduced by considering spatially extended systems.

### 2.1 General setting

Our model couples partial differential equations (PDEs), continuum models for diffusible substances, which drive individual cell behaviour, with a spatially discrete cell population dynamics described by a reaction–diffusion Master Equation (RDME) [68], [6], [38], [66], [33]. The scheme that we are using is a simple extension of the Chemical Master Equation (CME), where the space is discretised into a lattice. Each lattice site is associated to a compartment and the population within each compartment is assumed to be homogeneous (well-mixed) with reaction rates modelled according to the (local) law of mass action: reaction rates depend on the population within the corresponding compartment only. Diffusion is modelled by means of continuous-time random walk on the lattice.

Regarding the coupling between the diffusible substances (in this particular case, oxygen) and the birth-and-death dynamics, we assume that all the cells within a compartment are exposed to the same concentration and, therefore, they all respond identically to their stimuli. From the numerical point of view, we will assume that the grid on which we solve the PDEs is the same as the lattice which sustains the stochastic population dynamics. Since we primarily focus on one dimensional numerical experiments, PDEs are solved using a finite-difference discretisation and an explicit Euler or a four-stage Runge–Kutta method.

We must note that the RDME approach has been recently criticised as it has been shown that, except in one dimension, it does not converge to a continuum reaction–diffusion PDE [37]. A convergent RDME scheme has been recently proposed [38], although at this point we limit ourselves to the classical RDME scheme.

### 2.2 Resource layer: dynamics of diffusible substances

**Fig. 2:**
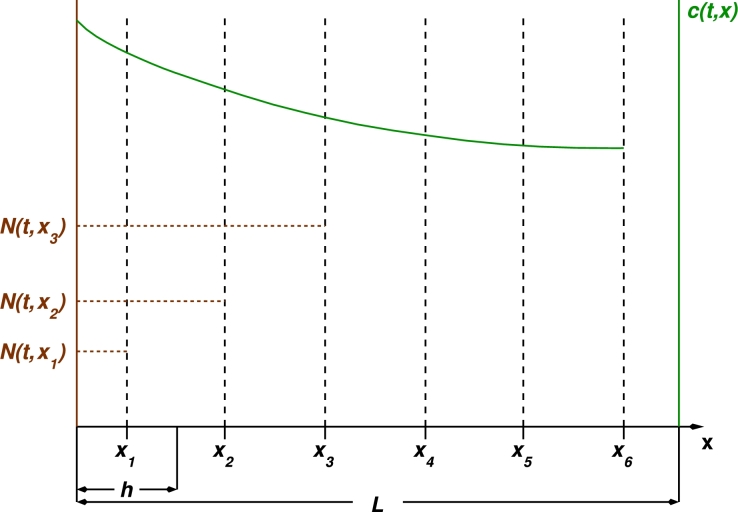
Representation of the setting of our model. Diffusible substances (e.g. oxygen), *c*(*t*,*x*), is modelled as a continuous field described by a reaction–diffusion PDE, it is represented by green solid line. The birth-and-death dynamics with diffusion of the cell population is modelled by means of a RDME on a lattice L. Each vertex of the lattice, $x_{i}\in L$, is associated to a compartment or voxel within which the population is assumed to be well-mixed and its stochastic dynamics ruled by a *local* law of mass action. *L* is the total length of the system and *h* is the lattice spacing, so that $L=N_{L}h$ where $N_{L}=\text{card}(L)$. Here *N*(*t*,*x*_*i*_) depict the number of cells in compartment *i* and it is calculated as $N(t,x_{i})=\int_{0}^{\infty }n(t,a,x_{i})da$, with *n*(*t*,*a*,*x*_*i*_) being the number of cells of age *a* at time *t* and compartment *x*_*i*_. (For interpretation of the references to colour in this figure legend, the reader is referred to the web version of this article.)

The evolution of the concentration of oxygen, c(t,x), (*resource scale*, see Fig. 1) is modelled by:

(1) $$\frac{\partial c}{\partial t}=D_{c}\frac{\partial^{2}c}{\partial x^{2}}-kc\sum_{x_{i}\in L}N(t,x_{i})\delta (x-x_{i})+S(t,x)-k_{2}c,$$

where *δ* is the Dirac delta function, L is the lattice defined in Section 2.1 (see Fig. 2), and $N(t,x_{i})$, $i=1,\ldots ,N_{L}$, is the number of cells in compartment *i* at time *t*. Note that, in general, the evolution of $N(t,x_{i})$ is a stochastic process, and, therefore, in principle Eq. (1) should be treated as a stochastic differential equation [35]. S(t,x) is a source that accounts for oxygen delivery to the system. $D_{c}$ is the oxygen diffusion coefficient, and *k* is the oxygen consumption rate. The term $k_{2}c$, has been included for two reasons. First, in its absence, the oxygen concentration ahead of the front grows boundlessly. This fact is likely to introduce artifacts in the stochastic population dynamics. Beyond that, this term also has a biological interpretation: it is associated with a native (passive) population which keeps the oxygen concentration finite, and against which the tumour modelled by our stochastic multi-scale system is growing.

### 2.3 Intracellular layer: oxygen-dependent birth rate

In our stochastic growth model, cell proliferation is fuelled by oxygen. The model of the oxygen-dependent growth rate is derived from a stochastic version of a model of oxygen-regulated progression through the cell cycle, in particular the G1/S transition [5]. The cell cycle is the pathway that orchestrates cell division. By means of this pathway, cells monitor their internal state and the presence of signalling cues. All this information is integrated by the cell cycle machinery which, upon detection of favourable conditions, drive the cell through a number of transitions between the different stages of the cell cycle (gap phase 1 (G1), DNA synthesis (S), gap phase 2 (G2), and mitosis (M)) which culminate with cell division at the end of the M-phase [26], [27], [28], [29], [30]. Mathematical modelling of the cell cycle has been traditionally done using systems of ordinary differential equations (ODEs) for the concentration of the proteins involved in cell cycle regulation [26]. Within this framework, the aforementioned transitions are identified with dynamically-induced bifurcations where key cell cycle regulators, known as cyclin dependent kinases (CDKs), are successively activated. Roughly speaking, the onset of each stage of the cell cycle is associated with the activation of the corresponding CDK [27]. Each stage therefore can be identified with the concentration of the associated CDK exceeding some threshold.

This picture lends itself to stochastic modelling, particularly to the application of first passage time problems, where one addresses the statistical properties of the time needed for the sample paths of a stochastic process to hit the boundary of a certain region. In this case, we are interested on the mean first-passage time (MFPT) problem associated to the activation of CycB (which binds to Cdk1), which determines the onset of the S-phase through the G1/S transition. The timing of this transition has been shown to strongly depend on the concentration of oxygen: the more abundant oxygen is, the faster the cell-cycle goes through the G1/S transition. Following our previous work [3], [51], [32], [17], we consider a simplified picture in which the time to the G1/S transition is regulated by a model of the oxygen-regulated G1/S transition and we lump the remaining of the cell cycle into an average waiting time. The biological justification for this comes from the fact that the duration of S, G2, and M seem to be only weakly dependent on the concentration of oxygen [2].

The MFPT associated to the stochastic version of the G1/S transition model proposed by Bedessem and Stephanou [5] has been studied in detail in [17]. Here, we report only the result of this analysis and refer the reader to our previous work for full details. We have shown that the MFPT for the cell to reach the G1/S transition depends on the oxygen concentration and the relative concentration of enzymes regulating the activity of SCF, an inhibitor of CycB [1], [16]. We refer to the MFPT for the cell to reach the G1/S transition as the age at the G1/S transition, $a_{G1/S}$, since this time is counted from the moment of birth of the cell:

(2) $$a_{G1/S}\left(c,\frac{p_{6}}{p_{3}}\right)≃\left\{ \begin{array}{c} a_{+}\left(\frac{p_{6}}{p_{3}}\right)e^{-c/c_{0}}\text{ if }\frac{p_{6}}{p_{3}}>r_{cr}\\ a_{-}{\left(\frac{c}{c_{cr}(p_{6}/p_{3})}-1\right)}^{-\beta }\text{ if }\frac{p_{6}}{p_{3}}<r_{cr}. \end{array}\right.$$

Here, $c_{0}$, $a_{\pm }$, and *β* are constants, $p_{6}$ and $p_{3}$ are the momenta coordinates associated with the SCF-activating and inactivating enzymes, $r_{cr}$ is critical value of the ratio $p_{6}/p_{3}$ above which there is no transition to quiescence, and $c_{cr}\left(\frac{p_{6}}{p_{3}}\right)$ is:

(3) $$c_{cr}\left(\frac{p_{6}}{p_{3}}\right)=1-\frac{1}{\beta_{1}}\log\left(\frac{1}{a_{3}H_{0}}\left(a_{1}+\frac{a_{2}d_{2}}{d_{1}{\left[e2f\right]}_{t}}\left(1-\frac{1}{1-a_{0}{\left(\frac{p_{3}}{p_{6}}\right)}^{2}}\right)\right)\right).$$

The parameters $a_{1}$, $a_{2}$, $a_{3}$, $d_{1}$, $d_{2}$, $\beta_{1}$, ${\left[e2f\right]}_{t}$, and $H_{0}$ are kinetic parameters of the mean-field model defined in [5] and [17] and $a_{0}$ is calculated in [17].

### 2.4 Cellular scale: age-structured birth-and-death with diffusion

**Fig. 3:**
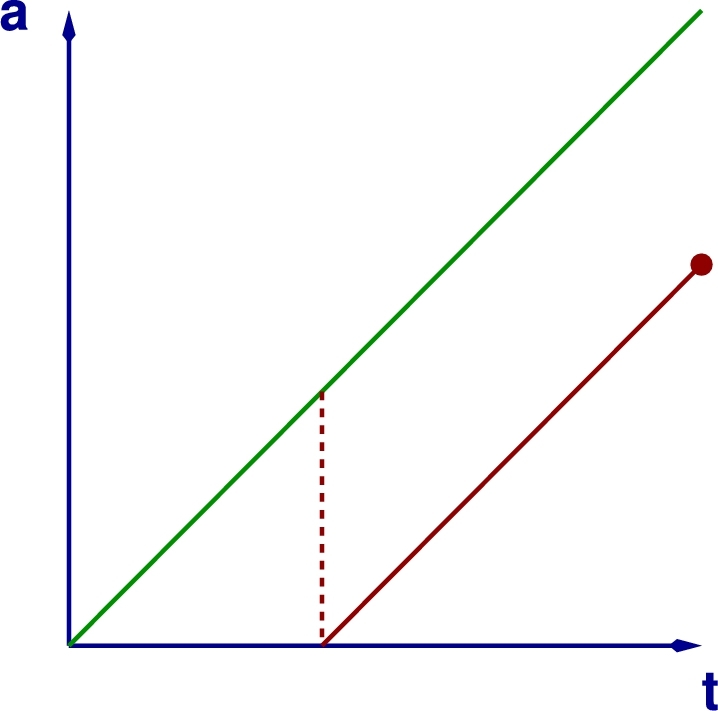
This plot shows a schematic representation of the characteristic curves, *a* = *t* + *a*_0_, corresponding to our age-structured stochastic dynamics and the emergence of new genealogies (red line) when a birth occurs (indicated by the red dashed line) within a previously existing one. Genealogies terminate when the corresponding population becomes extinct (indicated by the red point). (For interpretation of the references to colour in this figure legend, the reader is referred to the web version of this article.)

Consider the random variable $n(t,a,x_{j})$, i.e. the number of cells of age *a* in position $x_{j}$ at time *t*. Age is defined as the time elapsed since last division. Let us further define the random vector $N(t,a)=(n(t,a,x_{1}),\ldots ,n(t,a,x_{N_{L}}))$ where $N_{L}=\text{card}(L)$. An age-dependent Master Equation can be written for the birth-and-death with diffusion process described in Table 1, which is an extension of the age-dependent birth-and-death process formulated in [17] to account for the effects of cell diffusion. de la Cruz et al. [17] have shown that the evolution on each characteristic curve or *genealogy* (see Fig. 3) is independent and, therefore, the following equality holds on each characteristic curve:

(4) $$\begin{array}{ccc} P(N,t+\delta t,a+\delta a) & = & \sum_{j=1}^{N_{L}}\sum_{i=1}^{z+2}W_{i}(N-R_{i},t,a,x_{j})\delta tP(N-R_{i},t,a)\\ & & +\left(1-\sum_{i=1}^{4}W_{i}(N,t,a,x_{j})\delta t\right)P(N,t,a), \end{array}$$

where P(N,t,a) is the probability of the number of cells of age *a* to be N at time *t*, δa=δt, *z* is the coordination number of the lattice L and $R_{i}=(r_{i_{1}},\ldots ,r_{i_{N_{L}}})$. The quantities $W_{i}$, and $r_{i_{j}}$ are such that $P(N,a+\delta t,t+\delta t|N-r_{ij},a,t)=W_{i}(n,a,t,x_{j})\delta t+O(\delta t^{2})$, i.e. $W_{i}(n,a,t,x_{j})$ is the probability per unit time of event *i*, which can be birth, death or random diffusion (see Table 1), to affect the population $n(a,t,x_{j})$ in (t,t+δt). The vector $r_{ij}$ is the change in the state of the system associated with such event. Re-arranging terms and taking the limit δt→0, we obtain:

(5) $$\frac{\partial P(N,t,a)}{\partial t}+\frac{\partial P(N,t,a)}{\partial a}=\sum_{j=1}^{N_{L}}\sum_{i=1}^{z+2}\left(W_{i}(N-R_{i},t,a,x_{j})P(N-R_{i},t,a)-W_{i}(N,t,a,x_{j})P(N,t,a)\right).$$

**Table 1:** This table shows the details regarding the rates and stoichiometric matrix associated with the birth-and-death and diffusion process. This process is a straightforward generalisation of the age-dependent birth–death-process formulated in [17]. Here *b*(*a*) is the age-dependent birth rate, *ν* is the death rate (which, for simplicity, we assume to be age-independent), and *D*_*n*_ is the diffusion coefficient of the cells. *h* is the lattice spacing defined in Fig. 2. Furthermore, 〈*x*_*j*_〉 stands for the set of neighbours of *x*_*j*_ in L, and *z* is the so-called coordination number, i.e. the number of neighbours of a node: *z* = card(〈*x*_*j*_〉).

Event	Reaction	Transition rate, *W*_*k*_(*n*,*t*,*a*,*x*_*j*_)	*r*_*k*_
Birth	*n*(*t*,*a*,*x*_*j*_)→*n*(*t*,*a*,*x*_*j*_)−1	*W*_1_(*n*,*t*,*a*,*x*_*j*_)=*b*(*a*)*n*(*t*,*a*,*x*_*j*_)	$r_{1_{j}}=-1$, $r_{1_{k}}=0$, *k* ≠ *j*
	∅ → *n*(*t*,*a* = 0,*x*_*j*_)=2		

Death	*n*(*t*,*a*,*x*_*j*_)→*n*(*t*,*a*,*x*_*j*_)−1	*W*_2_(*n*,*t*,*a*,*x*_*j*_)=*νn*(*t*,*a*,*x*_*j*_)	$r_{2_{j}}=-1$, $r_{1_{k}}=0$, *k* ≠ *j*

Diffusion	*n*(*t*,*a*,*x*_*j*_)→*n*(*t*,*a*,*x*_*j*_)−1	$W_{2+k}(n,t,a,x_{j})=\frac{D_{n}}{h^{2}}n(t,a,x_{j})$, *k* = 1,…,*z*	$r_{2+k_{j}}=-1$, $r_{2+k_{l}}=1$, *x*_*l*_ ∈ 〈*x*_*j*_〉
	*n*(*t*,*a*,*x*_*j* ± 1_)→*n*(*t*,*a*,*x*_*j* ± 1_)+1		

A cornerstone in our model is the age-dependent birth rate, b(a), defined in Table 1, since it constitutes the coupling between the three layers that compose our multi-scale model: nutrient, intracellular, and cellular layer. In Section 2.3, we have discussed a model in which we consider that cell division is divided into parts: a regulated one culminating in the G1/S transition, characterised by the oxygen-dependent MFPT, $a_{G1/S}$, Eq. (2), and an unregulated one characterised by an average duration, $\tau_{p}^{-1}$. This model can be described by an age-dependent birth rate:

(6) $$b(a)=\tau_{p}^{-1}H(a-a_{G1/s}(c,p_{6}/p_{3})),$$

where H(⋅) is Heaviside's step function and $a_{G1/S}$ is given by Eq. (2). Eq. (6) can be interpreted as follows: cell division cannot occur in cells younger than the oxygen regulated age $a_{G1/S}$. Cells that have aged beyond $a_{G1/S}$ undergo cell division at a constant rate $\tau_{p}^{-1}$.

### 2.5 Linking scales together

The connections between different time-scales described in Fig. 1 is given by coupling the different sub-models so that the global behaviour of the system arises as an emergent property of this linkage:

1. The coupling between the intracellular and the cellular layers takes place through the age-dependent birth rate, Eq. (6).
2. The intracellular layer and the resource layer are coupled through the oxygen-dependence MFPT to the G1/S transition, given by Eq. (2).
3. Finally, the resource layer is coupled to the cellular layer, via Eq. (1), where the cell population regulates the concentration of oxygen, where *N* is
  (7) $$N(t,x_{j})=\int_{0}^{\infty }n(t,a,x_{j})da.$$

## 3 Separation of time scales and coarse-graining of the age structure

In this section we analyse the system regarding the separation of time scales in the system. This analysis requires to make assumptions involving typical key parameter values, particularly the oxygen diffusion coefficient, $D_{c}$, the cell diffusion coefficient, $D_{n}$, and the average cell life expectancy, $\nu^{-1}$. These assumptions are consistent with typical values of these parameters reported in the literature.

We start our analysis by considering the mean-field (deterministic) limit of our model, where the different time scales arise in a more intuitive way. We will then generalise our analysis to the full stochastic system using the Poisson representation [45], [4], [41], rather than the Master Equation representation Eq. (5). In both cases, we will show that provided that $\tau_{n}\gg \nu^{-1}\gg \tau_{c}$, where $\tau_{c}=h^{2}/D_{c}$ and $\tau_{n}=h^{2}/D_{n}$, is satisfied, it is possible to approximate the age-structured system by a coarse-grained version of it where the age distribution within the population reaches a quasi-steady state that can be explicitly calculated. This allows for a huge simplification of the model and the formulation of efficient hybrid numerical schemes.

### 3.1 Mean-field model

The mean-field limit of our stochastic model is a straightforward generalisation of the one obtained in [17] for the non-spatial model:

(8) $$\frac{\partial c}{\partial t}=D_{c}\frac{\partial^{2}c}{\partial x^{2}}-kN(t,x)c+S(t,x)-k_{2}c,\;N(t,x)=\int_{0}^{\infty }n(t,a,x)da,$$

(9) $$\frac{\partial n}{\partial t}+\frac{\partial n}{\partial a}=D_{n}\frac{\partial^{2}n}{\partial x^{2}}-(b(a)+\nu )n(t,a,x),$$

(10) $$n(t,a=0,x)=2\int_{0}^{\infty }b(a)n(t,a,x)da,$$

with no-flux boundary conditions at the boundaries of the domain and b(a) is given by Eq. (2). We first proceed to express the system in dimensionless units: τ=νt, α=νa, x→x/h, and $c\to c/c_{0}$, where $c_{0}$ is the characteristic scale of the oxygen concentration. Eqs. (8)–(10) now read:

(11) $$\epsilon_{1}\frac{\partial c}{\partial \tau }=\frac{\partial^{2}c}{\partial x^{2}}-\kappa N(\tau ,x)c+S(\tau ,x)-\kappa_{2}c,\;N(\tau ,x)=\nu^{-1}\int_{0}^{\infty }n(\tau ,\alpha ,x)d\alpha ,$$

(12) $$\epsilon_{1}\left(\frac{\partial n}{\partial \tau }+\frac{\partial n}{\partial \alpha }\right)=\epsilon_{2}\frac{\partial^{2}n}{\partial x^{2}}-(\tau_{c}b(\alpha )+\epsilon_{1})n(\tau ,\alpha ,x),$$

(13) $$n(\tau ,\alpha =0,x)=2\nu^{-1}\int_{0}^{\infty }b(\alpha )n(\tau ,\alpha ,x)d\alpha ,$$

with $\epsilon_{1}=\tau_{c}\nu \ll 1$, $\epsilon_{2}=\tau_{c}/\tau_{n}=D_{n}/D_{c}\ll 1$, $\kappa =\tau_{c}k$, and $S=\tau_{c}S/c_{0}$.

Under the assumption that $\epsilon_{1}\ll 1$, $\epsilon_{2}\ll 1$, and $\epsilon_{2}\ll \epsilon_{1}$ hold, we now show that there are three different regimes: a regime that corresponds to the very early evolution, where the oxygen distribution evolves under a constant-in-time population until it reaches a quasi-equilibrium state, an intermediate regime where the only evolution is associated to the local evolution of the age-distribution (no cell diffusion) until the age-distribution reaches a quasi-steady state equilibrium, and a third regime, which corresponds to the long-time evolution of the system, where both cell diffusion and population birth-and-death occur, only that the latter corresponds to the quasi-steady state age-distribution.

#### 3.1.1 Early evolution

Consider the following time and age re-scaling: $T=\epsilon_{1}^{-1}\tau$ and $A=\epsilon_{1}^{-1}\alpha$. Under this re-scaling the left-hand sides of Eqs. (11) and (12) become O(1). However, the two terms on the right-hand side of Eq. (12) stay $O(\epsilon_{2})$ and $O(\epsilon_{1})$. Therefore, at the lowest order, i.e. $O(\epsilon_{1}^{0})$, n(T,A,x)≃cnt., i.e. n(T,A,x)≃n(T=0,A,x). Therefore,

(14) $$\frac{\partial c}{\partial T}=\frac{\partial^{2}c}{\partial x^{2}}-\kappa N(x)c+S(T,x)-k_{2}c,\;N(x)=\tau_{c}\int_{0}^{\infty }n(T=0,A,x)dA,$$

that is the oxygen concentration evolves against a background of constant population, until it reaches a quasi-equilibrium oxygen distribution.

#### 3.1.2 Intermediate regime

This regime does not involve any re-scaling. We only need to recall that $\epsilon_{2}\ll \epsilon_{1}$ and $\epsilon_{1}\ll 1$. Under these assumptions, one can neglect $O(\epsilon_{2})$-cell diffusion in Eq. (12), with all the remaining terms of $O(\epsilon_{1})$. Similarly, we can neglect the $O(\epsilon_{1})$-left-hand side of Eq. (11) compared to the O(1) right-hand side. The resulting approximation reads:

(15) $$0=\frac{\partial^{2}c}{\partial x^{2}}-\kappa N(\tau ,x)c+S(\tau ,x)-\kappa_{2}c,\;N(\tau ,x)=\nu^{-1}\int_{0}^{\infty }n(\tau ,\alpha ,x)d\alpha ,$$

(16) $$\frac{\partial n}{\partial \tau }+\frac{\partial n}{\partial \alpha }=-(\nu^{-1}b(\alpha )+1)n(\tau ,\alpha ,x),$$

(17) $$n(\tau ,\alpha =0,x)=2\nu^{-1}\int_{0}^{\infty }b(\alpha )n(\tau ,\alpha ,x)d\alpha ,$$

this means the population evolves in a purely local fashion, with no diffusion, where within each compartment the age-distribution evolves towards equilibrium with a quasi-steady state oxygen distribution. This local quasi-equilibrium age-distribution has been studied and described in [17].

#### 3.1.3 Long-time behaviour

Consider the re-scaling $\sigma =\frac{\epsilon_{2}}{\epsilon_{1}}\tau$ and $\gamma =\frac{\epsilon_{2}}{\epsilon_{1}}\alpha$. Under this re-scaling, the left-hand sides of both Eqs. (11) and (12) become $O(\epsilon_{2})$. Since, the right-hand side of Eq. (11) remains O(1), the approximation is:

(18) $$0=\frac{\partial^{2}c}{\partial x^{2}}-\kappa N(\sigma ,x)c+S(\sigma ,x)-\kappa_{2}c,\;N(\sigma ,x)=\nu^{-1}\frac{\epsilon_{1}}{\epsilon_{2}}\int_{0}^{\infty }n(\sigma ,\gamma ,x)d\gamma ,$$

(19) $$\frac{\partial n}{\partial \sigma }+\frac{\partial n}{\partial \gamma }=\frac{\partial^{2}n}{\partial x^{2}}-\frac{\epsilon_{1}}{\epsilon_{2}}(\nu^{-1}b(\gamma )+1)n(\sigma ,\gamma ,x),$$

(20) $$n(\sigma ,\gamma =0,x)=2\nu^{-1}\frac{\epsilon_{1}}{\epsilon_{2}}\int_{0}^{\infty }b(\gamma )n(\sigma ,\gamma ,x)d\gamma .$$

Since $\epsilon_{2}\ll \epsilon_{1}$, Eqs. (19) and (20) imply that the local birth-and-death process, which drives the evolution of the age-distribution of the population, is much faster than the spatial spread of the cells. In other words, the local age-distribution reaches a quasi-equilibrium state against the background of a quasi-steady state distribution of oxygen. This property suggests that the long term evolution of the system can be described by a coarse-grained (age-independent) system where the local birth-and-death dynamics is determined by the (oxygen-dependent) net growth rate, $\lambda_{n}(c)$. This quantity can be obtained from the local equilibrium age distribution in a straightforward way, as we have shown in [17].

To make this statement more precise, consider a separable solution of Eqs. (19) and (20) in one dimension:

(21) n(σ,γ,x)=Σ(σ)Γ(γ)X(x),

and assume the oxygen concentration to be a constant. This solution is characterised by two sets of eigenvalues, $\lambda_{D}(k)$, which are associated to cell diffusion and which, for no-flow boundary conditions in one dimension, are given by $\lambda_{D}(k)=-k^{2}\frac{\pi^{2}}{L^{2}}$, k=0,1,2,… with *L* being the length of the domain measured in units of *h*, i.e. the lattice spacing. The second set of eigenvalues, $\lambda_{n}(c)$, are associated to the local birth-and-death dynamics. If b(a) is given by Eq. (6), $\lambda_{n_{k}}(c)$ are the solutions of the following characteristic equation [36]:

(22) $$2\frac{\epsilon_{1}}{\epsilon_{2}}{\left(\tau_{p}\nu \right)}^{-1}\frac{e^{-\left(\lambda_{n_{k}}+\lambda_{D}(k)+\frac{\epsilon_{1}}{\epsilon_{2}}\right)\gamma_{G1/S}}}{\lambda_{n_{k}}+\lambda_{D}(k)+\frac{1}{\epsilon_{2}}(\epsilon_{1}+\tau_{c}\tau_{p}^{-1})}=1,$$

where $\gamma_{G1/S}$ is a function of *c*. Now for each *k* we may solve for $\lambda_{n_{k}}$ in terms of $\lambda_{D}(k)$, allowing for a full expansion of the solution in terms of Fourier modes. That is, we extract the Fourier coefficients $a_{k},b_{k}$ from the Fourier expansion of the initial condition and we construct the associated solution as

(23) $$\begin{array}{ccc} n(\sigma ,\alpha ,x) & = & \Sigma (0)\Gamma (0)\sum_{k=0}^{\infty }e^{\lambda_{n_{k}}\sigma }\exp\left(\lambda_{D}(k)\gamma -(\epsilon_{1}/\epsilon_{2}+\lambda_{n_{k}})\gamma -\frac{\epsilon_{1}}{\nu \epsilon_{2}}\int_{0}^{\gamma }b(\tau )\;d\tau \right)\\ & & \times \left(a_{k}\sin(k\pi x/L)+b_{k}\cos(k\pi x/L)\right). \end{array}$$

Consider now the (unique) solution of Eq. (22) with $\lambda_{D}(k)=0$, $x_{0}$. Then $\lambda_{n_{k}}=x_{0}-k^{2}\frac{\pi^{2}}{L^{2}}$. Clearly the slowest mode would be the one for k=0. From Eq. (22) and since $\epsilon_{2}\ll \epsilon_{1}$, many modes have nearly the same time decay rate, being roughly the one dictated by $\lambda_{n_{0}}$. This accumulation of modes around the one associated to the equilibrium age-structure distribution justifies that the local age-distribution reaches a quasi-equilibrium state against the background of a quasi-steady state distribution of oxygen.

#### 3.1.4 Coarse-grained mean-field description

In view of the above analysis, particularly the results of Sections 3.1.2 and 3.1.3, we formulate a coarse-grained limit of Eqs. (11)–(13), where the local age-distribution is assumed to be in quasi-equilibrium with the quasi-steady distribution of oxygen:

(24) $$0=\frac{\partial^{2}c}{\partial x^{2}}-\kappa n_{cg}(\sigma ,x)c+S(\sigma ,x)-\kappa_{2}c,$$

(25) $$\frac{\partial n_{cg}}{\partial \sigma }=\frac{\partial^{2}n_{cg}}{\partial x^{2}}+\lambda_{n}(c)n_{cg},$$

(26) $$2\frac{\epsilon_{1}}{\epsilon_{2}}{\left(\tau_{p}\nu \right)}^{-1}\frac{e^{-\left(\lambda_{n}+\frac{\epsilon_{1}}{\epsilon_{2}}\right)\gamma_{G1/S}}}{\lambda_{n}+\frac{1}{\epsilon_{2}}(\epsilon_{1}+\tau_{c}\tau_{p}^{-1})}=1.$$

This description is valid from time σ=O(1) onwards. It will fail to describe the earlier evolution of the system, where the local age-distribution of the population has not had time to reach equilibrium.

### 3.2 Stochastic system

We now proceed with multiple time scale analysis of the full stochastic system. As in the case of the mean-field analysis carried out in Section 3.1, our aim here is to try and find regimes where the stochastic system defined in Section 2.4 can be approximated by simpler versions that are more amenable to analysis and efficient numerical simulation. In particular, we will show that, under the same hypothesis as in Section 3.1 regarding time scales, it is possible to coarse-grain the system so that the local (i.e. within each compartment) age-distribution of the population can be assumed to have reached quasi-equilibrium.

We start by reformulating Eq. (5) in terms of the Poisson representation [45], [31], [4], [41]:

(27) n(t,a,xj)=n(t=0,a−t,xj)−Y(∫0t(b(a(t))+ν)n(t,a(t),xj)dt)n(t,a,xj)=+∑xl∈〈xj〉(Y(τn−1∫0tn(t,a(t),xl)dt)−Y(τn−1∫0tn(t,a(t),xj)dt)),

(28) $$n(t,a=0,x_{j})=2Y\left(\int_{0}^{\infty }b(a)n(t,a,x_{j})da\right),$$

where a(t) is the equation of the characteristic curve (see Fig. 3) and Y(λ)∼Poisson(λ), i.e. Y(λ) is a random number sampled from a Poisson distribution with parameter *λ*. By writing the system in terms of the dimensionless variables τ=νt and α=νa:

(29) $$\epsilon_{1}\frac{\partial c}{\partial \tau }=\frac{\partial^{2}c}{\partial x^{2}}-\kappa c\sum_{x_{l}\in L}N(\tau ,x_{l})\delta (x-x_{l})+S(\tau ,x)-\kappa_{2}c,\;N(\tau ,x_{j})=\nu^{-1}\int_{0}^{\infty }n(\tau ,\alpha ,x_{j})d\alpha ,$$

(30) n(τ,α,xj)=n(τ=0,α−τ,xj)−Y(ϵ1−1∫0τ(τcb(α(t))+ϵ1)n(t,α(t),xj)dt)n(τ,α,xj)=+∑xl∈〈xj〉(Y(ϵ1−1ϵ2∫0τn(t,α(t),xl)dt)−Y(ϵ1−1ϵ2∫0τn(t,α(t),xj)dt)),n(τ,α=0,xj)=2Y(ϵ1−1∫0∞τcb(α)n(τ,α,xj)dα).

Under the same assumptions regarding time scales, i.e. $\epsilon_{1}\ll 1$, $\epsilon_{2}\ll 1$, and $\epsilon_{2}\ll \epsilon_{1}$, we now show that the stochastic system exhibits exactly the same three regimes as its mean-field counterpart, namely, early evolution, where the oxygen distribution evolves under a constant-in-time population until it reaches a quasi-equilibrium state, an intermediate stage where the local age-distribution evolves (with no cell diffusion) until it reaches a quasi-steady state equilibrium, and a third regime, associated with the long-time behaviour, where both cell diffusion and population birth-and-death occur with the latter determined by the quasi-steady state age-distribution.

#### 3.2.1 Early evolution

Consider the re-scaling: $T=\epsilon_{1}^{-1}\tau$ and $A=\epsilon_{1}^{-1}\alpha$. Under this change of variables, both the right and left-hand sides of Eq. (29) are now O(1). The stochastic evolution of the population becomes:

(31) n(T,A,xj)=n(T=0,A−T,xj)−Y(ϵ1∫0T(ν−1b(A(t))+1)n(T,A(t),xj)dt)n(T,A,xj)=+∑xl∈〈xj〉(Y(ϵ2∫0Tn(t,A(t),xl)dt)−Y(ϵ2∫0Tn(t,A(t),xj)dt)),n(T,A=0,xj)=2Y(ϵ1−1∫0∞ν−1b(A)n(T,A,xj)dA).

Taking into account that, upon re-scaling the waiting times associated to both diffusion and birth and death are very large, one can assume that $n(T,A,x_{j})=n(T=0,A-T,x_{j})+O(\epsilon_{1})$. So that at the lowest order, i.e. $O(\epsilon_{1}^{0})$, $n(T,A,x_{j})$ remains constant, $n(T,A,x_{j})≃n(T=0,A-T,x_{j})$ [4], [41], so that:

(32) $$\frac{\partial c}{\partial T}=\frac{\partial^{2}c}{\partial x^{2}}-\kappa c\sum_{x_{l}\in L}N(x)\delta (x-x_{l})+S(T,x)-\kappa_{2}c,\;N(x)=\nu^{-1}\epsilon_{1}\int_{0}^{\infty }n(T=0,A-T,x)dA.$$

#### 3.2.2 Intermediate regime

Since $\epsilon_{2}\ll 1$, to the lowest order, Eqs. (29) and (30) can be approximated by [4], [41]:

(33) $$0=\frac{\partial^{2}c}{\partial x^{2}}-\kappa c\sum_{x_{l}\in \langle x_{j}\rangle }N(\tau ,x)\delta (x-x_{l})+S(\tau ,x)-\kappa_{2}c,\;N(\tau ,x_{j})=\nu^{-1}\int_{0}^{\infty }n(\tau ,\alpha ,x_{j})d\alpha ,$$

(34) $$\begin{array}{c} n(\tau ,\alpha ,x_{j})=n(\tau =0,\alpha -\tau ,x_{j})-Y\left(\int_{0}^{\tau }(\nu^{-1}b(\alpha (t))+1)n(t,\alpha (t),x_{j})dt\right),\\ n(\tau ,\alpha =0,x_{j})=2Y\left(\nu^{-1}\int_{0}^{\infty }b(\alpha )n(\tau ,\alpha ,x_{j})d\alpha \right). \end{array}$$

Eqs. (33) and (34) imply that, during this intermediate dynamics, cell do not diffuse and the population dynamics is purely local against the background of a quasi-steady state distribution of oxygen. This evolution during this stage of the dynamics drives the local age-distribution towards local equilibrium.

#### 3.2.3 Long-time behaviour

We finish our analysis by, once again, considering the following re-scaling of the time and age variables: $\sigma =\frac{\epsilon_{2}}{\epsilon_{1}}\tau$ and $\gamma =\frac{\epsilon_{2}}{\epsilon_{1}}\alpha$. Under this change of variables and recalling that $\frac{\epsilon_{1}}{\epsilon_{2}}\gg 1$, Eqs. (29) and (30) transform into:

(35) $$0=\frac{\partial^{2}c}{\partial x^{2}}-\kappa c\sum_{x_{l}\in \langle x_{j}\rangle }N(\sigma ,x_{j})\delta (x-x_{l})+S(\sigma ,x)-\kappa_{2}c,\;N(\sigma ,x_{j})=\nu^{-1}\frac{\epsilon_{1}}{\epsilon_{2}}\int_{0}^{\infty }n(\sigma ,\gamma ,x_{j})d\gamma ,$$

(36) n(σ,γ,xj)=n(σ=0,γ−σ,xj)−Y(ϵ1ϵ2∫0σ(ν−1b(γ(t))+1)n(t,γ(t),xj)dt)n(σ,γ,xj)=+∑xl∈〈xj〉(Y(∫0σn(t,γ(t),xl)dt)−Y(∫0σn(t,γ(t),xj)dt)),n(σ,γ=0,xj)=2Y(ϵ1ϵ2∫0∞ν−1b(γ)n(σ,γ,xj)dγ),

where we have neglected terms of $O(\epsilon_{2})$.

Eqs. (35) and (36) are the basis for the formulation of our coarse-grained model. Eq. (36) implies that the rate at which the events associated with the local birth-and-death dynamics fire up is at a rate which is of order $O(\frac{\epsilon_{1}}{\epsilon_{2}})\gg 1$, whereas diffusion occurs at a rate of order O(1). This property implies that, between diffusion events and as long as such separation of time scales is large enough, the local age-distribution evolves through the birth-and-death dynamics, unperturbed by the diffusion part of the process, until it settles down onto quasi-equilibrium [10], [11].

#### 3.2.4 Stochastic coarse-grained model

On the basis of the argument we have put forward in Section 3.2.3, we propose a coarse-grained model of the stochastic evolution Eqs. (29)–(30). This coarse-graining strategy consists of integrating out the age structure taking and formulating the stochastic birth-and-death dynamics in terms of the total local population only. This is done by using the fact that age-distribution is in quasi-equilibrium, and therefore it is no longer valid in the early evolution of the system. In order to proceed with this programme, we first consider the number of new cells at time *σ* and position $x_{j}$, $n(\sigma ,\gamma =0,x_{j})$. According to Eqs. (6) and (36), we can write:

(37) $$n(\sigma ,\gamma =0,x_{j})=2Y\left(\frac{\epsilon_{1}}{\epsilon_{2}}{\left(\nu \tau_{p}\right)}^{-1}\int_{\gamma_{G1/S}}^{\infty }n(\sigma ,\gamma ,x_{j})d\gamma \right)=2Y\left(\frac{\epsilon_{1}}{\epsilon_{2}}BN(\sigma ,x_{j})\right),$$

where the coarse-grained birth rate is given by:

(38) $$B={\left(\nu \tau_{p}\right)}^{-1}\frac{\int_{\gamma_{G1/S}}^{\infty }n(\sigma ,\gamma ,x_{j})d\gamma }{\int_{0}^{\infty }n(\sigma ,\gamma ,x_{j})d\gamma }.$$

In a general setting, the quantity *B* should be considered as a function of time. However, under the conditions discussed in Section 3.2.3, where the age-distribution is in quasi-equilibrium, *B* is time independent. This is a simple consequence of the fact that at equilibrium, the ratio between the population younger than $\gamma_{G1/S}$ and the population older than $\gamma_{G1/S}$ is, on average, time independent [36].

Using the quasi-equilibrium condition, the (constant) fraction of the population younger than $\gamma_{G1/S}$ and the population older than $\gamma_{G1/S}$ can be estimated with regard to the probability of survival to age *γ*, $P_{S}(\gamma )$, which is given by:

(39) $$P_{S}(\gamma ,\lambda_{n})\propto e^{-\lambda_{n}\gamma -\frac{\epsilon_{1}}{\epsilon_{2}}\left(\gamma +{\left(\tau_{p}\nu \right)}^{-1}(\gamma -\gamma_{G1/S})H(\gamma -\gamma_{G1/S})\right)},$$

where $\lambda_{n}$ is the mean-field growth rate, given by the characteristic equation (Eq. (22)), and H(γ) is Heaviside's step function [36], [17]. At equilibrium, $P_{S}(\gamma ,x_{j})$ provides the proportion of the total population at compartment $x_{j}$ of age *γ*. Therefore the ratio in Eq. (38) can be calculated from Eq. (39), so that:

(40) $$B={\left(\nu \tau_{p}\right)}^{-1}\frac{\left(\lambda_{n}+\frac{\epsilon_{1}}{\epsilon_{2}}\right)e^{-\frac{\epsilon_{1}}{\epsilon_{2}}\gamma_{G1/S}}}{\lambda_{n}+\frac{\epsilon_{1}}{\epsilon_{2}}+{\left(\tau_{p}\nu \right)}^{-1}\frac{\epsilon_{1}}{\epsilon_{2}}\left(1-e^{-\frac{\epsilon_{1}}{\epsilon_{2}}\gamma_{G1/S}}\right)}.$$

Eq. (40) allows us to write a coarse-grained stochastic evolution which is given by:

(41) $$0=\frac{\partial^{2}c}{\partial x^{2}}-\kappa c\sum_{x_{l}\in \langle x_{j}\rangle }N(\sigma ,x_{j})\delta (x-x_{l})+S(\sigma ,x)-\kappa_{2}c,\;N(\sigma ,x_{j})=\nu^{-1}\frac{\epsilon_{1}}{\epsilon_{2}}\int_{0}^{\infty }n(\sigma ,\gamma ,x_{j})d\gamma ,$$

(42) Ncg(σ,xj)=Ncg(σ=0,xj)+Y(ϵ1ϵ2B∫0σNcg(t,xj)dt)−Y(ϵ1ϵ2∫0σNcg(t,xj)dt)Ncg(σ,xj)=+∑xl∈〈xj〉(Y(∫0σNcg(t,xl)dt)−Y(∫0σNcg(t,xj)dt)),

with *B* given by Eq. (40) taking into account that $\gamma_{G1/S}=\gamma_{G1/S}(c)$, i.e. it is a function of *c* given by Eq. (2).

## 4 Hybrid method for stochastic multi-scale models of tumour growth

We now proceed to describe the hybrid algorithm for stochastic multi-scale models. For simplicity, we focus our discussion to the case of a single interface in one dimension. The formulation of our hybrid methodology follows closely that of Spill et al. [67]. We consider an interface between the mean-field and stochastic domains based on the number of total number cells: the interface, *I*, is located in the last compartment such that its total population is larger than a threshold, Θ:

(43) $$N(t,x_{I})=\int_{0}^{\infty }n(t,a,x_{I})da>\Theta .$$

Since the description of the dynamics at the interface compartment is mixed, i.e. partly stochastic and partly mean-field, we impose that Θ be large enough compared to system size, so that both descriptions accurately account for the dynamics of the system. In order to increase the computational efficiency of our hybrid algorithm, we use the coarse-grained mean-field description coupled to the full age-structured stochastic model. We therefore need to provide specific rules for how age is introduced when cells are moved from the mean-field portion of the domain to the stochastic one.

*Hybrid algorithm*  We first provide a general overview of the algorithm. The parts that need more detailed discussion are dealt with later on.

1. Set Θ.
2. Set initial condition. We chose as an initial condition:
  $$N(t,x_{i})=KH(x_{I}-x_{i}),\;c(t,x_{i})=c_{\infty }=cnt.$$
  where *K* is the carrying capacity and $c_{\infty }$ is the associated equilibrium oxygen concentration. The age-distribution of the interface compartment, whose population dynamics is described by means of the stochastic model, is set to the equilibrium distribution Eq. (39). This choice is motivated by the fact that at the interface compartment both descriptions, i.e. the coarse-grained mean-field and the age-dependent stochastic process must hold.
3. Set time step as the waiting time to next stochastic event using the age-dependent Gillespie method [17].
4. Update the population within the stochastic domain according to the age-dependent Gillespie method [17].
5. Solve the PDEs for the coarse-grained mean-field population (over the mean-field domain) and the oxygen concentration (over the whole domain) in the interval [t,t+τ). The PDE for the oxygen is solved in the whole domain, mean-field plus stochastic parts, coupled to the coarse-grained population PDE and the stochastic population model, respectively.
6. Renormalise the number of cells in the interface compartment so that it is an integer.
7. Recalculate the position of the interface compartment.
8. Iterate 3–7 until some stopping condition is satisfied.

### 4.1 Coupling the mean-field and the stochastic models at the interface: fluxes, reactions and age-structure at the interface

This section provides details of how the mean-field and stochastic domains are coupled. Our procedure follows closely that presented in [67]. We currently provide the specific changes introduced to deal with age structure within the interface and beyond.

Recall that the interface compartment is considered as belonging to both the mean-field and the stochastic domains, so that the density of cells (associated with the mean-field description) is given by $n_{cg}(t,x_{I})=\frac{N(t,x_{I})}{h}$. Within the interface compartment, the population has age-structure and the birth-and-death dynamics is determined by the stochastic dynamics. The diffusive fluxes affecting it are considered exactly as in [67]: diffusion between the interface compartment and its stochastic neighbour are modelled using the usual diffusion transition rates:

(44) $$\begin{array}{c} W_{n_{x_{I}}-1,n_{x_{I}+1}+1|n_{x_{I}},n_{x_{I}+1}}=\frac{D}{h^{2}}n(a,t,x_{I}),\\ W_{n_{x_{I}}+1,n_{x_{I}+1}-1|n_{x_{I}},n_{x_{I}+1}}=\frac{D}{h^{2}}n(a,t,x_{I}+1), \end{array}$$

where, for simplicity, we have used the notation $n_{x_{I}}=n(a,t,x_{I})$. The diffusive fluxes to and from the last mean-field compartment need to be slightly modified with respect to the ones proposed in [67] in order to accommodate the age-structure of the interface population. The flux, $J_{I,I-1}$, from the interface compartment, $x_{I}$, into the last mean-field compartment, $x_{I-1}$, simply involves integrating out the age and using $n_{cg}(t,x_{I})=\frac{N(t,x_{I})}{h}$:

(45) $$J_{I,I-1}=\frac{D_{s}}{h^{2}}\left(n_{cg}(t,x_{I})-n_{cg}(t,x_{I-1})\right).$$

The flux from compartments $x_{I-1}$ into $x_{I}$ is modelled deterministically with $n_{cg}(t,x_{I})$:

(46) $$n_{cg}(t+\tau ,x_{I})=n_{cg}(t,x_{I})+\tau \frac{D_{s}}{h^{2}}\left(n_{cg}(t,x_{I})-n_{cg}(t,x_{I-1})\right).$$

We now need to address two issues: cells entering the interface compartment must be assigned an age and the number of cells must be an integer. In order to deal with these, we first renormalise the number of cells using the same procedure as in [67]. This procedure involves the consideration of the fractional part of the population probabilistically: we consider it as one minus the probability of regularising the interface population by removing its fractional part. In this case the excess of mass is moved to and distributed over the mean-field domain. Otherwise, the necessary amount of mass is evenly taken from the mean-field part and a whole cell is added to the interface. Upon renormalisation, we need to assign an age to the cell being removed or added to the interface. In the case of removal, this age is sampled from the age-structure distribution at the interface and one cell is removed from that age group. In the case of addition, the age of the added cell is sampled from the age-structure equilibrium distribution given by (39) and one cell is added to that age group.

### 4.2 Moving the interface

After completing the previous set of operations we must assess if the current interface position, held fixed through steps *3*–*6* of the hybrid algorithm, is consistent with the updated state of the system. If this is not the case then we shall relocate the interface accordingly before we resume with step *3*. Let us specify how to do this. First we check if the interface condition (43) continues to be satisfied at the interface compartment. If this is not the case (population at the current interface location dropped below Θ), we displace the interface one compartment to the left and check if the interface condition is met with this new choice of interface. If this happens to be true we stick with this new choice. If not we repeat this procedure until a compartment satisfying the interface condition is met, and then we displace the interface there. Incidentally we may find that we reach the left end of our spatial domain without fulfilling the previous criterion. In that case the whole spatial domain shall be described using the stochastic model on the next time step. However, this did not happen in any of our simulations – note that we are describing an invasion process.

Assume now that the interface condition (43) continues to be satisfied at the current interface compartment. What we do in this case is to check if the next compartment to the right does also satisfy this condition. In such a case we must shift the interface location one slot to the right and repeat this check (thus enlarging the mean field domain), until we make sure that we have relocated the interface so that its rightmost neighbour does not satisfy the interface condition.

The previous set of rules tells us how to position the interface after the state of the system is updated. If the interface experiences a net displacement then the mean field and stochastic regions are redefined. To be consistent with that we have to switch carefully between both descriptions at those voxels that changed from one domain to the other. If the interface moves to the right then we assign values to N(t,x) at those locations in which it was not previously defined simply by integrating out the age variable. The procedure is subtler if the interface moves left, as we have to provide an age structure to those compartments entering the stochastic region from scratch. In the simple case of the interface moving a single voxel to the left, we proceed as follows: (i) we convert population density to cell number at new interface's site – this will probably yield a non-integer cell number, (ii) we round the previous cell number into an integer value using the same mass transfer rules given in the previous paragraph, (iii) we assign an age structure to the resulting population by sampling the equilibrium age distribution (39) as many times as cells sit in the new interface compartment. Larger displacements are handled recursively by iterating unit displacements as we have just explained.

## 5 Assessing the accuracy of the coarse-grained and hybrid descriptions: travelling wave solutions

We now proceed to assess the accuracy of the coarse-grained mean-field and hybrid approximations by comparing them to the full stochastic simulations, which we take as our benchmark. We focus our analysis on the case where the system exhibits travelling wave behaviour. This regime has been used as a prototype to test a number of hybrid approaches in reaction–diffusion systems [67], [71]. Furthermore, travelling waves are a setting of particular interest regarding tumour modelling, as it has been used to describe both growth and invasion in several cancer models [25], [69]. In order to carry out a quantitative comparison between the predictions of the three models, we consider two quantities: the average position of the front of the travelling wave as a function of time, X(τ), and the average speed of the travelling wave.

**Fig. 4:**
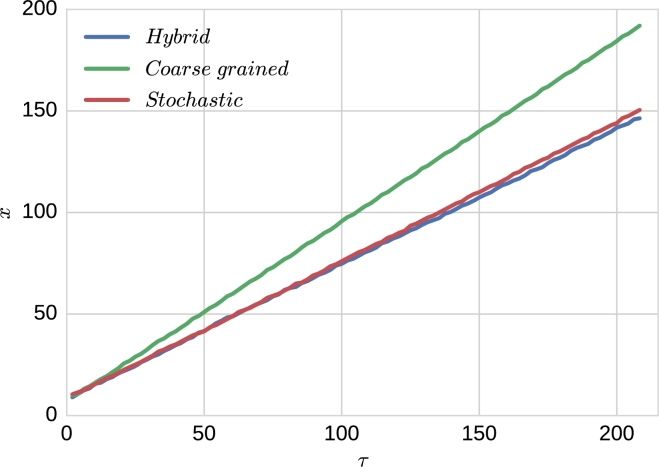
Plot showing the time evolution of the average front position for the three models: the coarse-grained mean-field model (green line), hybrid model (blue line), and stochastic model (red line). Results shown for the hybrid and stochastic models correspond to an average over 100 and 40 realisations respectively. Θ = 2000. For other parameter values see Section 5. Position calculation: average of the positions *x* where the population is greater than 0 and smaller than (K-100). (For interpretation of the references to colour in this figure legend, the reader is referred to the web version of this article.)

We start our discussion by comparing the (average) position of the wave front as a function of time. Fig. 4 shows that, for all three models, the system exhibits travelling wave behaviour as the position of the wave front is a linear function of time, i.e. their speeds are constants (see also supplementary movies A.8 and A.9). However, we appreciate that, whereas agreement between the results of the stochastic and hybrid models is rather good, an important departure exists between the results of the coarse-grained mean-field model and the stochastic and hybrid models.

**Fig. 5:**
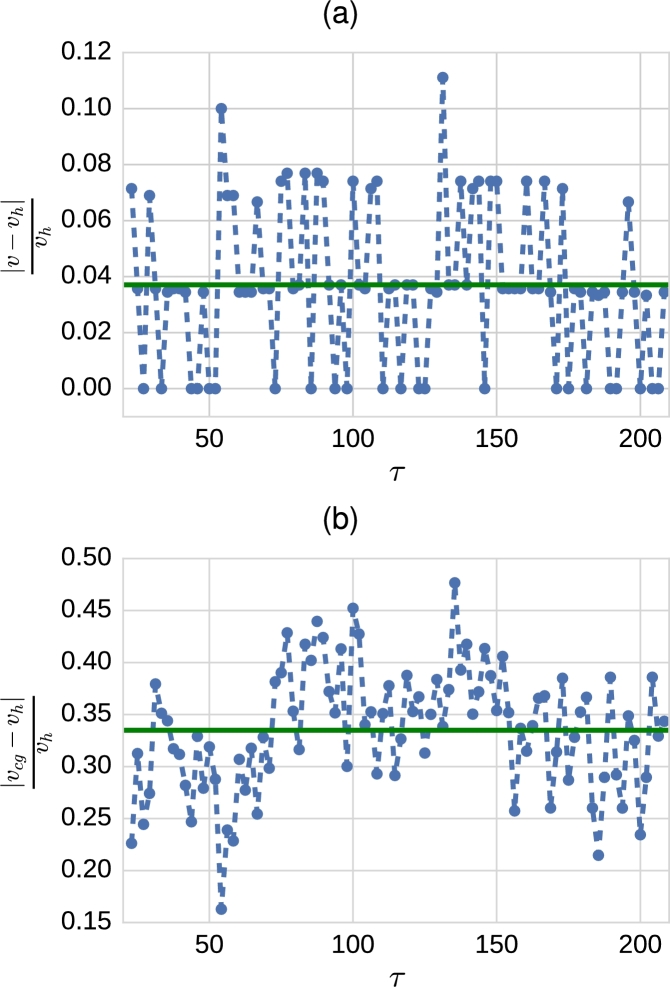
These plots show the absolute value of the relative difference between the velocity of the front predicted by hybrid simulations and full stochastic age-dependent SSA simulations (plot (a)) and the coarse-grained mean-field system (plot (b)). The green solid line represents the mean value over time. Each point corresponds to an average over 40 realisations of the age-structured SSA and 100 realisations of the hybrid method. The velocity of the front is calculated using the data corresponding to the position of the (average) front shown in Fig. 4. The threshold, Θ, in the hybrid simulations is taken to be Θ = 2000. (For interpretation of the references to colour in this figure legend, the reader is referred to the web version of this article.)

**Fig. 6:**
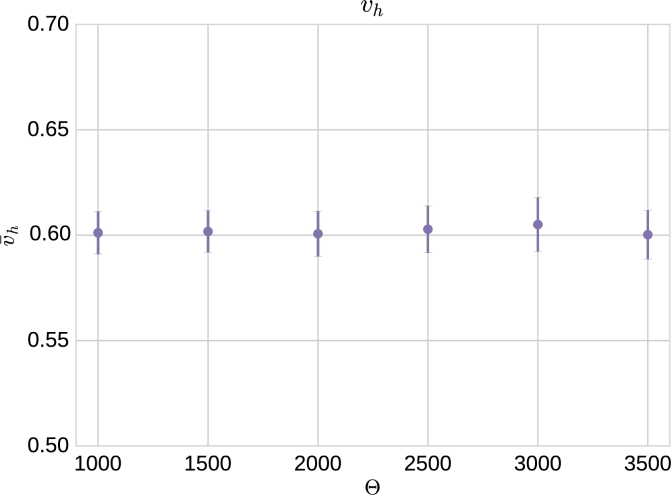
Comparison of the velocity average of 100 realisations of hybrid simulation with different threshold Θ = 1000,1500,2000,2500,3000 and 3500. Error bars correspond to standard error of the mean.

This discrepancy is analysed in a more quantitative way in the results shown in Fig. 5, where we compare the travelling wave velocity measured in the coarse-grained mean-field simulation, $v_{cg}$, the stochastic model simulations, *v*, and the hybrid method simulations, $v_{h}$. We observe that whereas relative difference between *v* and $v_{h}$ is typically of the order of a 5%, the relative difference between $v_{h}$ and $v_{cg}$ is of the order of a 30%. Before addressing this difference, we have checked the robustness of the wave speed $v_{h}$ with respect to variations in Θ. Our results, see Fig. 6, show that, as long as Θ is of the order of magnitude of the carrying capacity, *K*, $v_{h}$ is rather insensitive to changes in Θ (see also supplementary movie A.10).

The rather substantial difference observed in the wave speeds and how the hybrid model rectifies this variation need to be explained, since fluctuations in the $N(t,x_{j})$ do not seem to play a sufficiently significant role (see supplementary movie A.8). In order to address these issues, we need to look beyond purely *demographic* noise, i.e. fluctuations of $N(t,x_{j})$, and look into fluctuations associated with the age distribution of the population, or, in other words, fluctuations associated with the intracellular, cell-division dynamics. Recall that our coarse-grained approach is predicated upon the hypothesis that the age distribution of the system be in equilibrium (see Sections 3.1.3 and 3.2.3), given by Eq. (39). Under this hypothesis the growth rate of the population is given by Eq. (26), and the birth rate, whose general expression is Eq. (38), is given by Eq. (40). Departure of the empirically measured birth rate (i.e. using Eq. (38)) from its equilibrium value is a signature of non-equilibrium fluctuations of the age structure and, therefore, of the intracellular cell-division dynamics. It also heralds failure of the coarse-grained approximation to accurately describe the dynamics of the system.

**Fig. 7:**
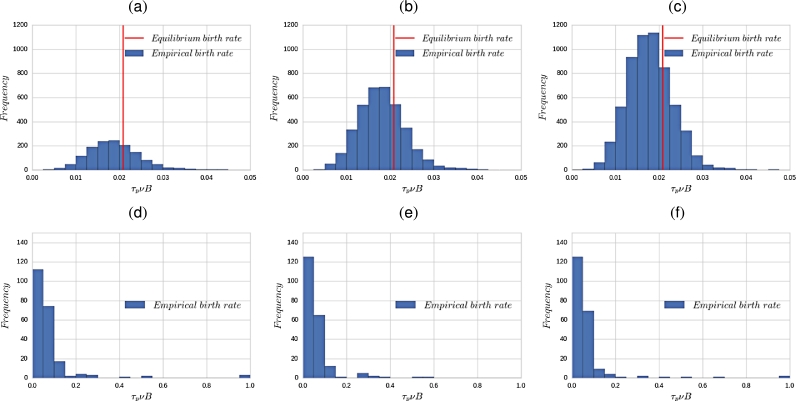
Plots showing the time evolution of probability distribution function of the birth rate as obtained from simulation of the full stochastic multi-scale model. We show three snapshots (time increasing from left to right) for the region behind the interface (upper row) and ahead of the interface (lower row). These results show that behind the interface the distribution of the empirical birth rate, calculated using Eq. (38), is centred around the equilibrium birth rate, Eq. (40), vertical red line. By contrast, ahead of the interface the birth rate distribution is much broader. Therefore, whereas behind the interface the equilibrium birth rate is a good approximation, this is not the case ahead of the interface. See also supplementary movies A.11 and A.12. Θ = 2000. For other parameter values see Section 5. (For interpretation of the references to colour in this figure legend, the reader is referred to the web version of this article.)

To assess the accuracy of the assumption that the age structure of the population is in equilibrium, we have run simulations of the full stochastic model and compared the empirical birth rate, Eq. (38), with equilibrium birth rate, Eq. (40). Results are shown in Fig. 7 and also in supplementary movies A.11 and A.12, where we plot the time evolution of the probability distribution function (PDF) of the empirical birth rate, as obtained from simulation of the full stochastic multi-scale model. We can see that behind the wave the front (Fig. 7(a), (b) and (c), and supplementary movie A.12(a)), where cell numbers approach the carrying capacity of the system, the empirical birth rate is reasonably approximated by the equilibrium birth rate. However, closer to and, crucially, ahead of the front, the empirical birth rate exhibits huge fluctuations both above and below the equilibrium birth rate. The latter implies that the age-structure of the population within the compartments located at the edge of wave is strongly off-equilibrium (Fig. 7(d), (e) and (f), and supplementary movies A.11 and A.12(b)). This observation explains the reason for the disparity of the wave velocities the coarse-grained mean-field and the stochastic models: the speed of propagation of a travelling wave is critically affected by the behaviour of the region close to the absorbing boundary, just at the edge of the wave front. In the case of our stochastic front, the equilibrium birth rate completely fails to describe the population dynamics in that region, and therefore the speed of the front is determined by stochastic effects associated to non-equilibrium fluctuations of the age distribution. By contrast, when the hybrid model is considered, the coarse-grained mean-field model deals only with the part of the system that is (approximately) in equilibrium. The part of the system whose age distribution is off-equilibrium, i.e. the population at the edge of the wave, is modelled by the full stochastic age-structured model, which provides the actual value of the birth rate. This fact also explains why the hybrid model provides a much more accurate description of the behaviour of the system.

*Parameter values*  The parameter values associated to the population dynamics are taken from [17]: $\nu =0.0000416667{\text{ min}}^{-1}$ and $\tau_{p}=480\text{ min}$. Estimates for the oxygen diffusion coefficient and the cell diffusion coefficient are $D_{c}=10^{-3}{\text{ mm}}^{2}/\text{sec}$ and $D_{n}=10^{-7}{\text{ mm}}^{2}/\text{sec}$, respectively [55], [63]. The rates of oxygen supply and oxygen consumption are taken to be $S=1.57\cdot 10^{-2}\text{ μM}/\text{sec}$ and $k=1.57\cdot 10^{-4}{\text{ sec}}^{-1}$ [17]. Furthermore, unless otherwise stated, the oxygen decay rate, $k_{2}$ is taken so that $\frac{S}{\kappa_{2}}=O(1)$. Finally, the carrying capacity, *K*, is given by:

(47) $$K=\frac{S}{\kappa c_{\infty }},$$

where $c_{\infty }$ is given by the (unique) solution of:

(48) $$\alpha_{G1/S}(c_{\infty },p_{6}/p_{3})=-\log\left(\frac{\tau_{p}\nu +1}{2}\right),$$

with $\alpha_{G1/S}(c_{\infty },p_{6}/p_{3})$ given by Eq. (2) [17], where we take $p_{6}/p_{3}=1$.

## 6 Discussion and conclusions

The development of hybrid methodologies is a current field of interest in both multi-scale modelling and stochastic reaction–diffusion systems, particularly regarding their applications to model biological systems. In this paper, we have proposed a hybrid methodology for a stochastic multi-scale model of tumour growth, i.e. a population-dynamical model which accounts for the effects of intrinsic noise affecting both the number of cells and the intracellular dynamics, associated in our model to cell proliferation (cell cycle). In order to formulate this method, we have developed an asymptotic theory which, taking into account the hierarchy of characteristic time scales and their separation, allows us to formulate a coarse-grained approximation for both the full stochastic model and its mean-field limit. This coarse-grained approximation involves averaging out the age-structure (which accounts for the multi-scale nature of the model) by assuming that the age distribution of the population settles onto equilibrium very fast compared with the time scales associated with cell motility.

Our hybrid model consists of coupling the coarse-grained mean-field model to the full stochastic simulation. By doing so, we are neglecting noise in both cell numbers (population) and their birth rates (structure). This means that, in addition to the issues that arise in stochastic-reaction diffusion systems, we need to account for the age-structure of the population when attempting to couple both descriptions. In this case, we exploit the nature of our coarse-graining strategy, namely, the fact that in the mean-field region the age-distribution is in equilibrium, of which we know its explicit form. This very much simplifies the coupling between both regimes, as upon transference of cells from the mean-field to the stochastic regime we just have to sample the equilibrium age distribution.

In order to check the accuracy of both the coarse-grained and the hybrid approximations, we have chosen to study a particular situation of interest in many biological problems, including tumour growth: the propagation of travelling waves. By taking as a benchmark the solution of the full stochastic model by means of the age-structured Gillespie algorithm previously formulated by de la Cruz et al. [17], we have been able to test both approaches for their accuracy in reproducing the behaviour of the moving front in terms of its position and velocity. The first observation we make is that the travelling wave velocity predicted by the mean-field coarse-grained model (where fluctuations in both population and birth rate are averaged out and thus not considered) is way off the benchmark. In fact, inaccuracies are larger than those expected from fluctuations in cell numbers alone. In view of this, we have investigated whether spatially-heterogeneous fluctuations of the birth rate are responsible for these discrepancies. Indeed, we have found that whereas such fluctuations have a modest effect behind the interface (i.e. in the mean-field region), noise associated to the birth rate is much larger ahead of the interface (i.e. in the stochastic region). The hybrid method, by incorporating the appropriate model of the birth rate in the different regions, leads to a much more faithful description of the dynamics of the full system than the coarse-grained limit alone.

We have thus formulated a method that extends the remit of existing hybrid methods for stochastic reaction–diffusion systems. A number of possible lines of improvement are shared with hybrid methods for reaction–diffusion systems: consistent ways to set the position of the interface (e.g. based on quantification of the local fluctuations), use of a convergent version of the Master Equation rather than the regular reaction–diffusion Master Equation, whose mean-field limit only converges to the associated reaction diffusion PDE in 1D [38], and extension to finite-element or unstructured meshes [21], among others. Other extensions of the current method are specific to the presence of structure variables, which reflect the multi-scale nature of the system. In particular, we need to explore the inclusion of more general structure variables (size, physiological, etc.) [18], [56], [57], for which the coarse-graining is likely to be more challenging. All these issues will be the subject matter of future research.

Our method has the additional merit of allowing us to explore the effects of intracellular noise, i.e. fluctuations in the birth rate associated with an out-of-equilibrium age distribution, on collective properties such as the speed of the travelling wave. We have showed that the interplay between population and structure noise results in large fluctuations of the birth rate in the region at the leading edge of front, which cannot be accounted for by the coarse-grained model. Such fluctuations have non-trivial effects on the speed of the wave. This leads us to conclude that the consideration of birth-rate fluctuations is necessary for a quantitatively accurate description of invasive phenomena such as tumour progression.

## Appendix A Supplementary material

Movie A.8 Simulation results showing the evolution of a travelling wave solution of the full stochastic model and the hybrid model. The results shown correspond to a single realisation. Θ = 2000. For other parameter values see Section 5. Movie A.8

Movie A.9 Simulation results showing the evolution of a travelling wave solution of the coarse-grained mean-field model and the hybrid model. Results shown for the hybrid correspond to a single realisation. Θ = 2000. For other parameter values see Section 5. Movie A.9

Movie A.10 Simulation results showing the evolution of a travelling wave solution of the hybrid model for different values of Θ. Results shown for the hybrid correspond to a single realisation and show that, within the range of values of Θ we have considered, the behaviour of the system depends very weakly on Θ. Parameter values are given in Section 5. Movie A.10

Movie A.11 Simulation results showing the comparison between the empirical birth rate, Eq. (38), and the equilibrium birth rate, Eq. (40). We observe that whereas behind the front of the wave both the latter approximates well the former, as we approach the edge of the wave the equilibrium birth rate fails to provide an accurate description of the empirical birth rate. The consequences of this result regarding the speed of propagation of the travelling wave are discussed in Section 5. Results shown for the stochastic model correspond to a single realisation. Θ = 2000. For other parameter values see Section 5. Movie A.11

Movie A.12a Simulation results showing the time dependent PDF of the birth rate behind the interface (movie (a)) and ahead of the interface (movie (b)). These results show that behind the interface the distribution of the empirical birth rate, calculated using Eq. (38), is centred around the equilibrium birth rate, Eq. (40), vertical red line. By contrast, ahead of the interface the birth rate distribution is much broader. Therefore, whereas behind the interface the equilibrium birth rate is a good approximation, this is not the case ahead of the interface. See also Movie A.11. Θ = 2000. For other parameter values see Section 5. Movie A.12a

Movie A.12b Simulation results showing the time dependent PDF of the birth rate behind the interface (movie (a)) and ahead of the interface (movie (b)). These results show that behind the interface the distribution of the empirical birth rate, calculated using Eq. (38), is centred around the equilibrium birth rate, Eq. (40), vertical red line. By contrast, ahead of the interface the birth rate distribution is much broader. Therefore, whereas behind the interface the equilibrium birth rate is a good approximation, this is not the case ahead of the interface. See also Movie A.11. Θ = 2000. For other parameter values see Section 5. Movie A.12b
